# Innovative humanitarian finance: examining the promises and realities of impact bonds in humanitarian and refugee response

**DOI:** 10.1111/disa.70074

**Published:** 2026-10-27

**Authors:** Laila Zulkaphil

**Affiliations:** ^1^ Humanitarian and Conflict Response Institute, The University of Manchester United Kingdom

**Keywords:** impact bonds, innovative humanitarian finance, outcome‐based finance, Payment by Results

## Abstract

This article contributes to scholarship on innovative humanitarian finance by examining the application of impact bonds—an outcome‐based financing mechanism where investors fund projects upfront and are repaid with interest if they succeed. Amid consistent funding shortfalls, impact bonds were framed as a radical transformation in how aid can be financed and delivered. By sharing financial risk with investors and linking payment to results, they were meant to attract private capital and enhance aid effectiveness. However, this study reveals a gap between the expectations and realities of impact bonds through case studies of two pilots: the Humanitarian Impact Bond (International Committee of the Red Cross); and the Refugee Impact Bond (Near East Foundation). In practice, impact bonds did not generate additional funds for the humanitarian sector, transfer significant risk to investors, or outperform traditionally financed programmes. The article therefore underscores the need for a rigorous evaluation of new financing mechanisms before adoption.

## 1 INTRODUCTION

Despite record levels of forced displacement and soaring needs, public funding of humanitarian assistance declined by 35 per cent in 2025 as compared to the previous year (OCHA, 2026). Recent aid cuts by major donor governments have exacerbated an already vast humanitarian funding gap. Over the past decade, rising humanitarian needs fuelled by violent conflicts, the climate crisis, and food insecurity have far outpaced available funding. In 2023, less than half of the total appeal coordinated by the United Nations (UN) was met, leaving a USD 30 billion shortfall—the largest recorded funding deficit to date (OCHA, 2025).

In this context, a consensus in the humanitarian sector holds that traditional funding models—especially short‐cycle grants from a small number of governments—are no longer adequate to meet the scale, duration, and complexity of today's crises (ODI, 2024). In response, the Grand Bargain—an agreement among humanitarian donors and agencies first adopted at the 2016 World Humanitarian Summit and later revised—called for mobilising resources from non‐traditional actors, such as the private sector and multinational banks, and for adopting innovative approaches to financing humanitarian action (IASC, 2016, 2023, 2024). Since then, various humanitarian actors, including the Danish Refugee Council, International Committee of the Red Cross (ICRC), International Federation of Red Cross and Red Crescent Societies, and International Rescue Committee, have piloted new financing instruments and approaches, such as impact bonds, disaster insurance schemes, refugee investment facilities, and technical advisory models.

There is a growing body of academic research (see, for example, Ahmed, 2021; Davitti, Arapiles, and Vidar, 2024; Davitti and Vankova, 2024, 2025; Shorey et al., 2024) and grey literature from think‐tanks (see, for example, Willitts‐King et al., 2019; Farber et al., 2024; ODI, 2024; Pullela and Velimirovic, 2025) exploring innovative, marked‐based financing instruments for humanitarian response. Still, there remains a need for more systematic inquiry that interrogates the assumptions underpinning these mechanisms and provides empirical evidence of whether they deliver on their stated promises. Consequently, this article seeks to contribute to building an evidence base for innovative humanitarian finance through an empirically grounded examination of one such mechanism: the use of impact bonds to fund humanitarian and refugee responses, focusing specifically on two pilot initiatives. It also aims to contribute to the broader impact bond literature by analysing the instrument's application in a new sphere: humanitarian and displacement settings.

An impact bond is an outcome‐based financing mechanism, wherein private investors provide upfront capital to service providers, and outcome funders repay the investors with interest if predefined outcomes are achieved. In 2016, the UN's High‐Level Panel on Humanitarian Financing (2016) recommended impact bonds as a promising means to unlock additional funding for the humanitarian sector, attract new types of donors, and develop a stronger results‐based culture. This article argues, based on a comparative case study of two pioneering applications in humanitarian and displacement settings, that impact bonds in practice have fallen short of such aspirations. The cases examined are the ICRC's Humanitarian Impact Bond (HIB), which provided physical rehabilitation services for conflict‐affected communities in Africa, and the Refugee Impact Bond (RIB) implemented by the Near East Foundation (NEF) to support the livelihoods of Syrian refugees and host communities in Jordan. The article draws on empirical data from 30 interviews with actors involved in the aforementioned endeavours, other humanitarian professionals who have explored impact bonds, and independent experts, alongside an analysis of more than 50 documents pertaining to the two cases.

Section two of this article traces the origins and evolution of impact bonds, beginning with Social Impact Bonds (SIBs) and their derivative, Development Impact Bonds (DIBs), before appraising their nascent application within the humanitarian sector. Section three outlines the research methodology, explaining the rationale for the case selection and detailing the data collection methods. Section four examines the two cases against the main promises associated with impact bonds. Although they were introduced as a potential solution to the humanitarian funding gap, this section demonstrates that impact bonds did not generate new resources. Instead, they reallocated existing funds, with investors advancing capital that traditional donors later reimbursed. Section five interrogates the claim that impact bonds enhance aid effectiveness by shifting the focus from inputs to outcomes. Evidence of improved outcomes is lacking in the cases studied, with impact bonds either not designed in a manner to drive enhanced outcomes for aid recipients or delivering results no better than traditionally financed programmes. Section six investigates the claim that impact bonds function as a risk‐sharing mechanism that fosters programme innovation. However, the study shows that they did not transfer significant or distinctive risks to investors that traditional humanitarian donors would be unwilling or unable to bear. The article concludes by highlighting in section seven the importance of rigorously assessing new financing tools to ensure their application is fit for purpose.

## 2 OVERVIEW OF IMPACT BONDS

### 2.1 Impact bonds: the concept and origins

Impact bonds are a type of outcome‐based financing instrument, also known as ‘Payment by Results’ (PbR), where payment is conditional upon the achievement of predefined outcomes. Outcome‐based financing is contrasted with input‐based financing, whereby a funding agency prefinances an implementer to deliver a project and assumes the risk of failure. Grants, which are commonly used to fund aid programmes, follow the logic of input‐based financing. Although grant‐funded programmes work towards specific outcomes and are evaluated against key performance indicators, in what is known as ‘results‐based management’, funds are allocated based on planned activities or inputs rather than disbursed after the verification of achieved outcomes. A key argument for outcome‐based financing is that switching the conditions of payment from activities to outcomes shifts service provider behaviour and improves social outcomes for target populations (Economy, Carter, and Airoldi, 2023).

In conventional PbR arrangements, service providers bear financial risk by pre‐financing projects using their own resources or bank loans (Economy, Carter, and Airoldi, 2023). What differentiates impact bonds—their primary innovation—is the participation of investors, who cover the upfront costs of projects and receive a return on their capital depending on results (Broccardo, Mazzuca, and Frigotto, 2020; Economy, Carter, and Airoldi, 2023). This structure is designed to transfer risk away from both funding agencies and service providers. Impact bonds, therefore, convene three main parties: (i) investors—individuals or investment firms; (ii) outcome funders—governments or philanthropic foundations; and (iii) implementing organisations, also known as service providers. Beyond these entities, intermediary actors play a pivotal role, such as by conducting feasibility studies, facilitating negotiations among the parties, and supporting performance management (Liang, Mansberger, and Spieler, 2014; Gustafsson‐Wright et al., 2017). Most impact bonds also engage independent evaluators to verify whether outcomes have been met, technical consultants to advise on outcome metrics and payment terms, and legal counsels to draft and review contracts (Gustafsson‐Wright et al., 2017).

Although the concept of SIBs was proposed by a New Zealand economist in 1988, it was not until the global financial crisis of 2008 that they garnered significant attention (Joy and Shields, 2017). In an era of fiscal austerity, governments began exploring SIBs as a cost‐saving mechanism since they were designed to finance preventative and early intervention programmes targeting costly social problems such as prison recidivism, homelessness, and chronic health conditions—areas with the potential for a long‐term reduction of public expenditure (Liang, Mansberger, and Spieler, 2014). Rather than delivering or directly funding such programmes, governments would raise private capital from investors to finance external service providers to deliver them. If the programmes achieve predefined outcomes, the government repays the investors plus a return on their capital; if outcomes are not met, the investors bear the cost fully or partially. Such framing of public spending as contingent upon success was appealing to both budget‐constrained governments and taxpayers (Liang, Mansberger, and Spieler, 2014). Despite the limited success of initial pilots in the United Kingdom and the United States aimed at reducing reoffending among prisoners, both governments continued to promote SIBs, committing substantial funding to their expansion (Liang, Mansberger, and Spieler, 2014; Joy and Shields, 2017; Dey and Gibbon, 2018). While the UK endorsed SIBs as a cost‐saving tool, the US positioned them as a means to test innovative approaches and redirect funding towards ‘what works’—a strategy enabled by the transfer of financial risk to investors and the model's emphasis on outcome‐based flexibility (Albertson et al., 2018).

As of May 2025, 259 impact bonds have been contracted across 40 countries, spanning sectors such as social welfare, employment, education, health, and criminal justice (Gustafsson‐Wright and Painter, 2025). While the majority of impact bonds are concentrated in high‐income countries, 34 have been implemented in low‐ and middle‐income countries. Of the latter, 19 are classified as DIBs. The key difference between SIBs and DIBs lies in who pays for outcomes: under SIBs, the outcome funder is the national or local government, guaranteeing repayment for domestic programmes if outcomes are met, whereas DIBs involve third‐party funders such as foreign donor governments, philanthropic foundations, or multilateral institutions. DIBs are deployed where SIBs are infeasible, either because the state lacks the resources to pay for outcomes or fails to inspire investor confidence (Alenda‐Demoutiez, 2020).

### 2.2 Impact bonds in humanitarian contexts

In the early 2010s, some donors, such as the UK and the World Bank, began experimenting with PbR in international development programmes, driven by the logic that linking funding to actual results would enhance accountability and improve performance (Guerrero‐Ruiz, 2025). The UK, in particular, drew on its extensive domestic experience of PbR and sought to transfer these practices to the Department for International Development (DFID) owing to a growing incentive to demonstrate value for money as overall aid budgets tightened (DFID, 2014). By the late 2010s, other donors, including Norway, Switzerland, and the European Union, had also adopted PbR, establishing it as a standard instrument within the development financing toolkit (Guerrero‐Ruiz, 2025). Efforts have also been made to push PbR further up the results chain, tying payments to longer‐term outcomes rather than short‐term outputs, thereby moving closer to genuine outcome‐based financing (DFID, 2014). In this context, impact bonds emerged as a relevant instrument, allowing for longer‐term outcome measurement, while the provision of upfront capital by investors broadens access to outcome‐based financing for smaller organisations that lack liquidity and the capacity to absorb risk (DFID, 2014).

In the humanitarian sector, however, the uptake of PbR has been limited and remains largely experimental. Crisis‐affected environments are seen as too volatile and high risk for outcome‐based financing, given the strong influence of external factors beyond the control of service providers (Interviewees 2 and 23).^1^ When the overriding imperative is to save lives, the administrative demands of data collection and third‐party verification are perceived as secondary (Interviewee 1). These considerations are compounded by the practical challenges of obtaining reliable outcome‐level data in rapidly changing contexts, particularly where populations are mobile (Interviewees 15 and 21). As a result, input‐based financing remains the default instrument in humanitarian response, as it is considered faster, simpler, and more appropriate for rapid‐onset emergencies (Interviewees 18 and 23). Nonetheless, there is growing recognition that outcome‐based financing holds promise in protracted crises and displacement settings to support ‘nexus’ programming (Interviewees 17 and 22), which is precisely where the two cases examined here—the HIB and the RIB—were developed.

The emergence of the humanitarian–development nexus has challenged the assumption that humanitarian and development interventions operate in distinct spheres. The link between humanitarianism and development was previously understood as a linear ‘continuum’, with development assistance following humanitarian relief once an emergency subsides (Macrae et al., 1997; Macrae and Harmer, 2004; Levine and Mosel, 2014). By contrast, the nexus approach is based on a ‘contiguum’ model, which conceives humanitarian and development actions as taking place simultaneously and pursuing shared goals; some interpretations extend this logic to a ‘triple nexus’ that incorporates peacebuilding (Schmitz Guinote, 2019; Bradley, 2023). This shift reflects the reality of increasingly protracted and complex crises involving a mixture of conflict, displacement, state failure, violence against civilians, and populations in chronic vulnerability (Donini, 1998; Barnett, 2005). Greater coherence between humanitarian and development actions is intended to mitigate emergencies by reducing people's vulnerability to shocks (Macrae and Harmer, 2004; Hilhorst, 2018), while also serving donors' implicit aim of containing displaced populations within their regions of origin and curbing onward migration (Zetter, 2021). Impact bonds, with their multi‐year horizons and emphasis on measurable and durable outcomes, align with nexus thinking.

At the same time, the widening humanitarian funding gap—from well before the 2025 government aid cuts and the dismantling of the United States Agency for International Development—prompted humanitarian organisations to seek resources from different actors, particularly the private sector (Girling‐Morris, 2022; ODI, 2024; Pearson, Girling‐Morris, and Nelson‐Pollard, 2025). In this context, the UN's High‐Level Panel on Humanitarian Financing (2016) recommended impact bonds as a (promising) mechanism that would open up new avenues of financing for the humanitarian sector, attract new types of donors, and develop a stronger results‐based culture. Subsequently, the ICRC and NEF set up impact bonds targeting populations affected by conflict and displacement.

Despite being one of several innovative financing mechanisms tested in the humanitarian sector and representing a small share of the wider landscape of humanitarian finance, impact bonds have attracted considerable attention. ICRC President Peter Maurer announced the launch of the HIB at the 2016 World Economic Forum in Davos, Switzerland, attended by heads of state and global corporate leaders, and the initiative soon made headlines in mainstream media such as *The Economist* (2017). The RIB also received significant attention in humanitarian and development circles as the world's first refugee‐focused impact bond (Willitts‐King et al., 2019; Convergence, 2024). Since then, uptake of impact bonds in the humanitarian sector has been limited, but new initiatives, such as the Syria Humanitarian Mine Action and Livelihood Development Impact Bond, are under development (Humanitarian Finance Forum, 2025), suggesting continued interest in the instrument and underscoring the need for an empirical study of the early cases.

Launched in 2017, the ICRC's HIB was a five‐year programme that involved building three physical rehabilitation centres in the Democratic Republic of the Congo (DRC), Mali, and Nigeria, and operating them more efficiently as compared to the historic average of comparable centres (Ecorys, 2022). To this end, the ICRC tested and implemented Efficiency Improvement Measures (EIMs), deployed a Digital Centre Management System (DCMS) to enhance care and resource management, and built local capacity through staff training and government collaboration (Farber and Huang, 2022a, 2022b; Grant et al., 2022). German reinsurance company Munich Re, Swiss banking group Lombard Odier, and other private sources invested CHF 18.6 million to prefinance the aforementioned activities. Outcome funders, including the governments of Switzerland, Belgium, Italy, and the UK, as well as the La Caixa Foundation, committed to paying the investors up to CHF 26.1 million, including a maximum seven per cent annual return on their investment if the programme demonstrated an 80 per cent improvement against the outcome metric (Lau, 2021). If the new centres operated less efficiently than comparable centres or did not open at all, the investors would still recoup 60 per cent of their principal (Ecorys, 2022). When the HIB concluded in July 2022, achievement against the outcome metric, known as the Staff Efficiency Ratio (SER), was 1.09, meaning that the three new centres operated on average nine per cent more efficiently than baseline centres (Grant et al., 2022). Although the achievement did not yield a return on investment, the investors recovered their full principal.

The four‐year RIB launched in October 2021 was set up to help Syrian refugees and vulnerable host community members in Jordan meet their basic needs through micro‐enterprise creation (Borkum et al., 2022). It was born out of a feasibility study conducted in 2016–17 by KOIS Invest, a Belgian firm specialising in impact investing, to explore innovative financing solutions for the Syrian refugee crisis. Later, KOIS selected NEF as the implementing partner of the RIB. The U.S. International Development Finance Corporation and Ferd, a family‐owned Norwegian investment company, invested USD 9.8 million. The outcome funders, the IKEA Foundation, the Novo Nordisk Foundation, and the Norwegian Agency for Development Cooperation (Norad), committed a maximum of USD 13.8 million, including repayment to investors with an annual interest of 5.1 per cent (IKEA Foundation et al., 2021; Bery, 2023). The programme outcome was measured against two metrics: (i) the survival rate of businesses created; and (ii) households' consumption rate (Borkum et al., 2022). If the programme failed to achieve its targets against the outcome metrics, the investors would lose 20 per cent of their principal, with the remaining 80 per cent guaranteed regardless of outcomes (IKEA Foundation et al., 2021). When the programme ended in October 2025, it had surpassed the ceiling for maximum investor payments pertaining to the business survival metric, which accounted for most of the returns, and met the minimum threshold for payments linked to the household consumption metric (Meyer, Borkum, and Collins, 2025).

## 3 METHODOLOGY

Methodologically, the article employs a qualitative, comparative case study approach. It treats impact bonds as a case of innovative humanitarian finance, since they are part of a wider set of new financing instruments designed to leverage government or philanthropic funds to attract private capital to the fragile contexts of humanitarian action. Within this broader framing, the HIB and the RIB serve as embedded cases that illustrate how impact bonds have been adapted to humanitarian and displacement settings. These two initiatives were selected because, at the time of the research, they were the first and only impact bonds implemented in humanitarian environments and evaluated by third parties, making them information‐rich cases capable of generating analytically valuable insights. They also represent contrasting organisational traditions, enabling a meaningful comparison. The ICRC, as a humanitarian actor whose mandate is rooted in international humanitarian law, exemplifies ‘classical’ humanitarianism characterised by strict adherence to humanitarian principles (Labbé and Daudin, 2015). By contrast, NEF, originally established as Near East *Relief* in 1919 to provide emergency aid to Armenian refugees in the Ottoman Empire, rebranded itself in 1929 as the Near East *Foundation* to indicate a shift from short‐term relief towards longer‐term self‐reliance of refugees and host communities (Rodogno, 2022). This orientation aligns with ‘new’ or ‘resilience’ humanitarianism, which seeks to move beyond alleviating immediate suffering towards building the long‐term resilience of affected populations (Fox, 2001; Hilhorst, 2018). Therefore, a comparative study facilitates a richer understanding of how impact bonds interact with different humanitarian logics.

Primary data were collected through key informant interviews (KIIs). Sampling involved two complementary strategies: quota sampling; and snowball sampling. As impact bonds involve different types of actors—including service providers, investors, outcome funders, and intermediaries—quota sampling ensured the interviewing of a minimum number of informants from each category. Additionally, snowball sampling was employed by asking the interviewees to recommend others with the required background or knowledge. KIIs are broken down by category in Table 1.

**TABLE 1:** KIIs by category.

Key informants by category	Number of interviews
Humanitarian professionals	8
Independent experts	8
Investors	5
Intermediaries	5
Outcome funders	4
**Total**	**30**

**Source:** author.

Six interviewees were directly involved in the HIB in various capacities and another six were involved in the RIB. The remaining participants, although not directly linked to either case, possessed relevant knowledge of and insights into impact bonds. Such participants included humanitarian professionals who explored impact bonds as part of their organisational fundraising strategies, investors and intermediaries involved in other DIBs, and experts from research institutions and think‐tanks who had studied impact bonds, including the two cases examined here. The interviews followed a semi‐structured format, informed by an interview guide but adapted to each participant's expertise and to emerging lines of inquiry. Implementers, investors, outcome funders, and intermediaries were asked different sets of questions reflecting their distinct responsibilities and perspectives. For interviewees not directly involved in the two cases, questions focused first on their own research on or professional experience of impact bonds, and then on the specific cases if the interviewees were familiar with them. Given that participants were geographically dispersed, the interviews were conducted online via a video‐conferencing platform and lasted for approximately one hour. They took place over four months from February to May 2024 after ethics board approval was obtained. Verbal informed consent was acquired from all participants prior to each interview, including agreement to recording, use of anonymised data, and inclusion of anonymous quotations. Interviewees are referred to by numbered pseudonyms (1, 2, 3, etcetera) in a consistent but non‐identifying manner.

Document analysis constituted an additional data collection method and served several functions. Documents provided contextual and background information essential for understanding each case, supported the identification of key informants, and offered supplementary empirical material. In particular, they supplied factual details, such as programmatic and financial information, that informed interview preparation and helped to ensure that interview time was used efficiently. They also functioned as an important source of triangulation. Convergence between data obtained through KIIs and document analysis enhanced confidence in the credibility of the findings, whereas any inconsistencies prompted further investigation. Triangulation also entailed comparing documents produced by actors directly involved in the cases with those generated by unaffiliated sources, recognising that such materials are created for particular audiences and purposes and should not be taken as direct reflections of reality (Yin, 2014).

A total of 51 documents were analysed, encompassing a range of materials, such as news articles, evaluation reports, case studies, web pages, and press releases. They were identified through a snowballing web‐search strategy, in which sources cited in initial materials were examined and included when relevant. Some interviewees also recommended or directly shared documents with the author. Of those reviewed, 28 pertained to the HIB, 16 focused on the RIB, and 6 either discussed other impact bonds or offered general insights relevant to the study. More documents pertaining to the HIB were identified because it concluded earlier, producing more publicly available learnings, and because it attracted significant attention as the world's first humanitarian impact bond implemented by a major humanitarian actor. Together, the KIIs and the document analysis formed the empirical foundation for the findings presented in the following sections.

## 4 DID IMPACT BONDS GENERATE NEW FUNDING?

The rationale for setting up the HIB and the RIB was to mobilise additional and diversified funds to address growing humanitarian needs and the widening funding gap (Maurer, 2017; PR Newswire Africa, 2017; Parker, 2019; IKEA Foundation et al., 2021; Adamkiewicz, 2022; ICRC, 2022). In particular, the ICRC's publicly stated motivation for launching the HIB was to diversify its funding base beyond annual voluntary contributions by governments and to access private funds (Bollag, 2017; Ecorys, 2022; Farber and Huang, 2023). If successful, the HIB would not only enable the ICRC to gain invaluable expertise in innovative finance but also ‘open up a new source of funding for the humanitarian sector as a whole’ (Farber and Huang, 2022a, p. 12). Similarly, the RIB was presented as a solution to both funding and programmatic challenges; it would mobilise additional resources for the underfunded context of the Syrian refugee crisis while supporting more effective programmes that improve the livelihoods of refugees and host communities (Interviewee 15; see also IKEA Foundation et al., 2021; Near East Foundation, 2022).

Although humanitarian actors pursued impact bonds with the expectation of securing additional and diversified funds for their organisations and the wider humanitarian sector, they were not an effective means of achieving this goal. This belief stems from viewing impact bonds as a *fundraising* tool, when in reality, they function as a *financing* mechanism. One expert (Interviewee 25) noted that *funding* and *financing* are often conflated when speaking of innovative humanitarian finance, explaining the difference using the example of a mortgage: a mortgage from the bank is a type of *financing* to enable the purchase of a house while one's income is a source of *funding* that ultimately pays for the house. Similarly, the initial capital raised from investors through impact bonds is *financing*, not *funding*. It is a loan to prefinance activities until actual *funding* becomes available upon completion of the programme, contingent on the achievement of outcomes.

Since investor capital does not represent additional funding, the question of whether the HIB brought new resources to the ICRC is more appropriately assessed through the outcome funders. The only new donor to the ICRC was the La Caixa Foundation, the smallest among the outcome funders, contributing CHF 1 million out of the CHF 26.1 million outcome fund (Lau, 2021). The other outcome funders—namely, the governments of Switzerland, the UK, Belgium, and Italy—were already part of the ICRC's traditional donor base (ICRC, 2026). In fact, the ICRC intentionally worked with its existing donors on the HIB as it deemed navigating a new financing mechanism and new relationships simultaneously would be too challenging (Farber and Huang, 2023).

Although the UK was an existing donor to the ICRC, its funding for the HIB (CHF 2.5 million) came from a budget specifically designated for testing impact bonds in overseas programmes funded by the Foreign, Commonwealth, & Development Office (FCDO). Since this budget was not sector‐specific, it remained separate from its humanitarian funding (Interviewee 17). In contrast, Switzerland's contribution to the HIB—the largest outcome funder, contributing CHF 10 million—was drawn from the Swiss Agency for Development and Cooperation's humanitarian budget, which would likely have been allocated to the ICRC or a similar humanitarian agency regardless of the financing mechanism (Interviewee 14). The other outcome funders seem to have taken a similar approach. A review of ICRC *Annual Reports* indicates that between 2016 (the year before the HIB launched) and 2022 (the year it concluded), annual contributions from Belgium and Italy remained steady (see Figure 1). The finding suggests that the HIB did not generate additional funds from these donors, at least not significantly, and ultimately played a limited role in expanding and diversifying funding for the ICRC or the humanitarian sector.

**FIGURE 1:**
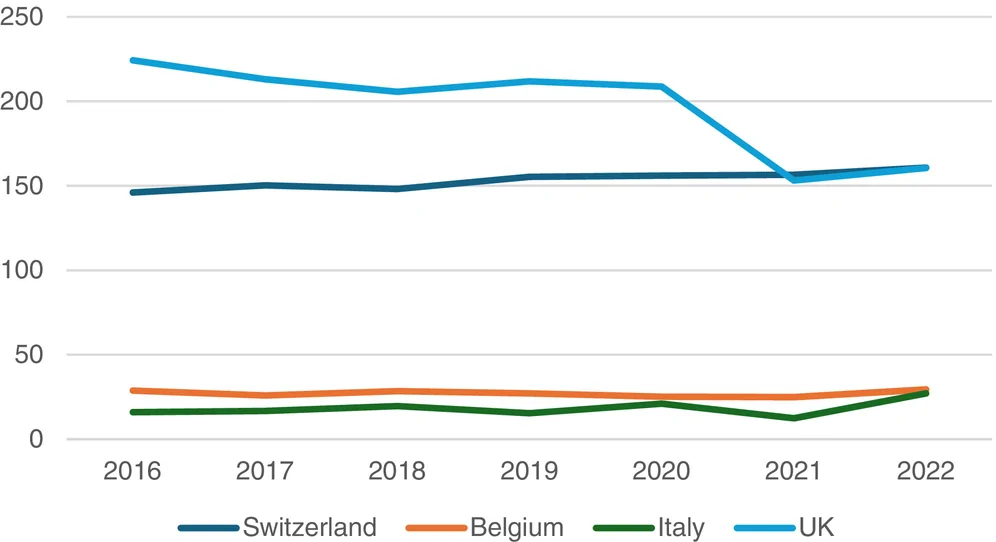
HIB outcome funders' annual contributions to the ICRC in millions of CHF. **Source:** author, based on ICRC (2026).

In contrast, the RIB appears to have supported NEF's objective of securing additional and diversified resources. The RIB expanded the organisation's livelihood programming in Jordan, and all three outcome funders—the IKEA Foundation, the Novo Nordisk Foundation, and Norad—were new donors to NEF (Interviewees 5 and 20; see also Redvers, 2021). However, they were already established players in humanitarian and refugee response. For instance, both the IKEA and the Novo Nordisk Foundations had funded similar programmes to support the livelihoods of Syrian refugees and host communities in Jordan (Malik et al., 2018; Novo Nordisk Foundation, 2019).

As the outcome funders in both cases were established donors in humanitarian and displacement contexts, the HIB and the RIB did not generate additional funding beyond what was already likely to be available. More broadly, the availability of new resources depends on donors' interests and their willingness to support a specific intervention, regardless of the financing mechanism used.

While impact bonds can mobilise additional funding for individual organisations, as the RIB did for NEF, they are far from a straightforward funding solution owing to the significant time and resources required for setup. The HIB took three years to move from the design stage (2014) to its launch in 2017 (Farber and Huang, 2023), whereas the RIB required five years from the feasibility study (2016) to its launch in 2021 (Convergence, 2017; Bery, 2023). One interviewee explained: ‘Don't think that this is a simple way of getting funds…I'd be very careful in terms of not having wrong illusions from the start of how complex it is’ (Interviewee 8). Another interviewee emphasised that treating impact bonds as a distinct funding stream is misguided: ‘The fund‐seeking route is not a route that focuses on the additional impact and added value that the impact bond can have, and they often will find that traditional mechanisms are easier to get that funding in place’ (Interviewee 18).

Furthermore, the assumption that impact bonds reduce costs by shifting the burden of unsuccessful outcomes to investors—while donors pay only for success—has not been substantiated in practice. Outside the humanitarian sector, of the nearly 50 impact bonds completed as of 2020, programme targets were reached and investors were paid back in all but two cases (Gustafsson‐Wright, Massey, and Osborne, 2020). Investors interviewed for this study corroborated this high success rate, reporting that they have recouped at least their principal in almost all impact bond contracts in which they have participated (Interviewees 10 and 12). Even if programmes fail to reach performance targets, substantial guarantees frequently embedded in the design of impact bonds ensure that investors are able to recoup most of their principal. For example, as noted, 80 per cent of investors' principal in the RIB and 60 per cent in the HIB were guaranteed regardless of actual outcomes. Outcome funders are also committed to programme success, motivated not only by reputational risks associated with high‐profile pilots, but also by financial systems that struggle to reabsorb committed funds from prior budget cycles when outcomes are not achieved.

No new money enters the humanitarian sector through impact bonds when outcome funders are established humanitarian donors; in fact, money exits the humanitarian sector. As one interviewee said: ‘There is obviously a sort of leakage of money which would go outside of the humanitarian environment, say USD 100 arrive and a certain percent goes to the private sector because you outsource some risk’ (Interviewee 8). The ‘leakage of money’ is not limited to the premium paid to investors. Setting up and managing impact bonds results in significant additional expenditures, such as feasibility studies, intermediary services, and legal and financial advisory services, which typically would not be incurred in conventionally financed programmes. The FCDO‐commissioned evaluation of four DIBs, including the ICRC's HIB, found that additional costs were in the range of USD 1.8–2.3 million; these ranged from 9–42 per cent as a share of the total programme budget (Grant et al., 2022).

Rather than bringing in new money, impact bonds primarily changed the flow of money. Instead of paying for programmes upfront, donors paid in arrears based on outcomes achieved, while implementing organisations received the liquidity needed from investors. However, unlike frontloading mechanisms such as vaccine bonds issued by the International Finance Facility for Immunisation (Gavi: The Vaccine Alliance, 2025) or similar models explored for humanitarian mine action (Loughran, Payne, and Wallen, 2024), which are explicitly designed to bring future donor commitments forward so that large amounts of funding become available immediately, the HIB and the RIB were not intended to serve this function. Instead of making long‐term pledges of future aid, outcome funders paid into an escrow account in advance to assure investors that the contracted outcome payments would be honoured. In the case of the RIB, outcome funders contributed to the escrow annually (Interviewee 16), whereas in the HIB case they made a one‐time payment of the maximum possible amount (Farber and Huang, 2023). As a result, neither instrument functioned as a frontloading mechanism in practice, even though impact bonds are sometimes described as a way to unlock liquidity that would otherwise be unavailable. In both instances, the outcome funding was already secured in the escrow account and could, in principle, have been used to finance programme activities.

## 5 DID IMPACT BONDS IMPROVE OUTCOMES?

A central premise of outcome‐based financing is that paying for results rather than inputs can yield greater impact by shifting the focus from activities to outcomes and incentivising stronger performance. If impact bonds demonstrably improve humanitarian outcomes for affected populations, they add value even when they do not mobilise additional resources or frontload liquidity. Accordingly, this section assesses whether the HIB and the RIB led to improved outcomes relative to conventional financing models.

The HIB's outcome metric was designed to compare the SER of the three new physical rehabilitation centres in the DRC, Mali, and Nigeria with that of four existing ICRC centres in Guinea‐Bissau, Niger, South Sudan, and Sudan (Farber and Huang, 2022b). If the HIB centres had achieved an 80 per cent efficiency gain over the comparator sites, the investors would have earned the maximum annual return of seven per cent, with proportionately lower returns for lesser achievements (Farber and Huang, 2023). However, the new centres operated only nine per cent more efficiently on average than the baseline, which, as noted, was not sufficient to trigger a financial return for the investors; therefore, the investors received their principal back without any profit (Grant et al., 2022). The slightly higher achievement as compared to the baseline was only because of the centre in the DRC (SER = 1.54); the centres in Nigeria and Mali did not demonstrate any improvement (SER = 0.80 and 0.67, respectively) (Ecorys, 2022). Before the new centres became operational, the ICRC identified efficiency improvement measures and tested them in eight existing physical rehabilitation centres, which improved their SER by 30 per cent (Ecorys, 2022). Since the non‐HIB centres performed more efficiently under the same measures, and two of the three new centres demonstrated no improvement, the impact bond mechanism overall did not yield better outcomes than the ICRC's traditional financing model.

The HIB's outcome was affected by multiple external factors, including the COVID‐19 (coronavirus disease 2019) pandemic, delays in mobilising personnel for the new centres, and security constraints, such as the Boko Haram insurgency in Nigeria and two coups d'état in Mali (Ecorys, 2022; Farber and Huang, 2022c; Grant et al., 2022). Consequently, the centres became fully operational only in the final year of the five‐year programme. The majority of the programme's duration was consumed by infrastructure development and preparatory activities, leaving limited time and scope to implement the intended efficiency‐enhancing interventions. Ultimately, though, these challenges suggest a degree of misalignment between the financing mechanism and programme design. Outcome‐based financing models such as impact bonds are suitable when the pathway to achieving desired outcomes is uncertain (Interviewees 10, 16, and 21). Specifically, the flexibility of outcome‐based financing models as compared to more rigid grants allows organisations to implement programme activities in an iterative and adaptive manner and to test different approaches to identify what works. Moreover, the risk transferred from donors should be dependent on human behaviour—of those delivering or receiving services—rather than external risks which implementing organisations cannot influence (Interviewee 23). In the case of the HIB, the construction of new centres and the development of digital systems followed a linear work schedule rather than iterative design or experimentation. Additionally, the primary risks to performance stemmed from external operational factors, rather than the behaviour of service providers or end users. As such interventions do not benefit from the outcome‐focused flexibility and behavioural incentives that impact bonds are designed to support, it would have been more appropriate for donors to fund them directly through traditional financing models without incurring a premium or creating additional complexity.

Of the programme's components, only the effort to boost efficiency met the criteria of iterative experimentation and dependence on human behaviour—specifically, staff productivity. However, the chosen outcome metric, the SER—defined as ‘the number of mobility devices delivered per physical rehabilitation professional’ (Farber and Huang, 2022b, p. 8)—overlooks vital dimensions of aid effectiveness, including patient satisfaction, service quality, and total number of people served (Ecorys, 2022). Hence, the HIB's outcome metric offered little insight into how well the programme met humanitarian needs in terms of quality of care and overall reach. As one interviewee explained, the outcome metric had to be simple and easily measurable to eliminate any ambiguity as it had significant financial implications (Interviewee 8). Therefore, the metric appears to have been designed primarily to satisfy the financial structure of an impact bond, to determine payment triggers and investor returns. These limitations indicate that the HIB did not deliver on its promise to shift the focus from inputs and outputs to outcomes.^2^ What was dubbed an ‘outcome metric’ still concentrated on outputs (mobility devices) and inputs (staff time).

Similarly, the ICRC's longstanding physical rehabilitation programme was chosen not on a needs basis but because it had robust baseline data, a multi‐year focus suitable for an impact bond, and easily measurable gains in mobility (Interviewees 8 and 13; see also Bollag, 2017; *The Economist*, 2017; Lau, 2021; Farber and Huang, 2022b). The programme had ‘strong evidence for the approach’ (Lau, 2021, p. 12) and was ‘venerated both within the ICRC and beyond…for the impact it delivered’ (Farber and Huang, 2023, p. 6). This is not to say that the programme had no room for improvement, but rather that the need to improve outcomes was not the main driver for financing this programme through an impact bond.

Whereas the HIB relied on a single operational efficiency metric that did not adequately capture the programme's impact on aid recipients, the RIB employed a two‐tier outcome framework, comprising business survival and household consumption rates, with the latter measured through quasi‐experimental treatment and control groups. The RIB demonstrated mixed performance across these metrics. In relation to the business survival metric, the RIB substantially exceeded the 55 per cent minimum target and 75 per cent cap for maximum investor payments: third‐party evaluators verified that 98 per cent of beneficiaries had an active business 10 months after receiving business grants (Meyer, Borkum, and Collins, 2025). Although one interviewee suggested that the 60‐day reference period for verifying a business's most recent transaction was too generous and made the target easy to meet, the average number of days since the last sale was eight and the median was three, indicating that most businesses were making frequent transactions (Interviewee 16; see also Meyer, Borkum, and Collins, 2025).

The second metric, however, yielded more modest results. The annual household consumption (relating to food and non‐food goods and services) of the treatment group was JOD 636 higher than that of the control group, corresponding to an effect size of 0.225 standard deviations. This result was sufficient to trigger investor returns, but it was considered ‘similar or slightly lower’ than comparable livelihood and cash interventions in Jordan and Lebanon (Meyer, Borkum, and Collins, 2024, p. 6). The evaluation employed a modified formulation of the household consumption metric, diverging from the original framework by using aggregate household consumption rather than per capita figures, and excluding debt repayment owing to concerns about data reliability. Had the original definition been retained, the evaluation would have shown no statistically significant difference between the treatment and control groups (Meyer, Borkum, and Collins, 2024, p. 10). Moreover, the increase in consumption was uneven across groups and tended to benefit those who were already relatively advantaged. For instance, Jordanian households recorded an average consumption gain of JOD 858 relative to the control group, whereas refugee households saw only a marginal gain of JOD 214 (Meyer, Borkum, and Collins, 2025). Thus, while the RIB achieved strong short‐term results for business survival, it did not lead to substantial improvements in longer‐term livelihood outcomes when compared with traditionally financed interventions in the region, especially for refugee households, despite the instrument's explicit focus on them.

Interviewees attributed the positive results of the RIB, particularly in relation to the business survival metric, to agile and adaptive programme management, which typically is not possible under more restrictive grant funding (Interviewees 5, 6, and 16). NEF had flexibility to change the implementation plan and budget as needed to achieve the outcomes and respond to real‐time needs rather than merely implementing the activities as described in donor‐approved documents. Stronger monitoring systems and analytical tools adopted under the RIB allowed the programme team to identify challenges and course‐correct in a timely manner. In addition to adaptive management, the RIB provided multi‐year financial stability, enabling the team to concentrate more deeply on implementation without having to fundraise and plan for subsequent projects (Interviewee 6). This sustained funding supported institutional learning through a multi‐cohort approach, enhanced engagement with recipients, and helped to improve the quality of programming (Interviewees 5 and 16). By contrast, similar benefits were not observed in the ICRC case. Whereas NEF's field team in Jordan was afforded the autonomy to drive implementation and the flexibility to adapt activities as needed, ‘field staff on the ground in each of the HIB locations were limited in their ability to be flexible, as overall project and performance management were held by HQ [headquarters] in Geneva’ (Ecorys, 2022, p. 21). Moreover, as the HIB centres were operational for only a year, there was insufficient time to incorporate learning and adapt implementation (Grant et al., 2022). Although the ICRC also benefitted from having predictable, longer‐term funding (Interviewee 24; see also *The Economist*, 2017), this feature was not used to iterate and adapt interventions and improve outcomes as expected under an outcome‐based financing model; rather, it was used for long‐term infrastructure investments such as building new centres and systems.

Overall, the evidence shows that neither impact bond delivered stronger outcomes than comparable traditionally financed interventions. The HIB's efficiency gains were limited by both external constraints and a rigid programme design ill‐suited for an impact bond. The RIB used the instrument more flexibly to adapt and iterate, which contributed to strong business survival, but its impact on household consumption was more limited. These findings resonate with the existing literature on SIBs, which questions whether the additional costs and complexities of impact bonds are justifiable in the absence of robust evidence that they deliver better outcomes than alternatives. Although most impact bonds achieve their stated outcomes and pay returns to investors, this success alone does not confirm the mechanism's effectiveness in the absence of a counterfactual—evidence that similar outcomes would not have occurred without the impact bond (Fraser et al., 2018, 2020; Gustafsson‐Wright, Massey, and Osborne, 2020; Economy, Carter, and Airoldi, 2023). Some studies have compared SIB‐funded programmes with similar programmes financed using other instruments (Wong et al., 2016; Dayson, Fraser, and Lowe, 2020; Carter, 2021), but these are too few and small scale to draw a conclusion on the effectiveness of impact bonds. Furthermore, payment conditions are not always tied to genuine outcome‐level changes, and rigorous evaluation methods such as randomised controlled trials are seldom employed (Gustafsson‐Wright, Massey, and Osborne, 2020). Critics argue, therefore, that impact bonds could lead to perverse incentives to select the easiest outcomes, encouraging service providers to prioritise short‐term gains over addressing deep‐rooted social problems, while outcome funders risk paying a premium for results that would have occurred without the impact bond (Broccardo, Mazzuca, and Frigotto, 2020; Economy, Carter, and Airoldi, 2023).

## 6 DID IMPACT BONDS TRANSFER RISK?

In addition to serving as an outcome‐based financing model, impact bonds are often presented as a mechanism for sharing risk with investors. This feature is particularly relevant in situations where outcome funders or implementing agencies may be unwilling or unable to shoulder substantial risks, such as when piloting innovative interventions with limited prior evidence or expanding small‐scale interventions with initial promising evidence (Interviewees 7, 10, 18, 23, and 27). The complexity and costs of impact bonds make them unsuitable for well‐established programmes or ‘business as usual’; traditional financing instruments are more efficient for funding such proven approaches (Interviewees 7, 18, and 21). Accordingly, this section examines whether the two cases under review involved innovation or performance risk that went beyond the routine operational uncertainties typically encountered in humanitarian programming.

Whether the HIB enabled innovative and risky interventions that could not have been funded otherwise is questionable. The ICRC runs physical rehabilitation centres all over the world, operating 139 in 34 countries at the time of setting up the HIB in 2016 (*The Economist*, 2017; Farber and Huang, 2022a). As one interviewee shared, other than the conflict‐related and political risks that are present in most contexts in which the ICRC operates, the HIB did not present any unique performance risks warranting investor involvement (Interviewee 2). Similarly, another interviewee remarked that donors should have directly funded an infrastructure intervention that the ICRC knows how to do and has done before, without introducing a complex mechanism that involves investors (Interviewee 21).

According to the ICRC, the HIB introduced innovations through experimental tools such as the DCMS and EIMs, which would have been challenging to fund outside of the efficiency‐focused HIB (Lau, 2021; Grant et al., 2022). Yet, these new elements were *process* innovations focusing on how efficiently the centres used staff time and raw materials and how they tracked and managed data. The HIB lacked *programme* innovation, as no new interventions or approaches were introduced to improve outcomes for aid recipients. In outcome‐based financing models, innovation and flexibility should be applied higher up the results chain to drive better outcomes for recipients, rather than incremental improvements related to processes (DFID, 2014). Furthermore, according to the FCDO‐commissioned evaluation by Ecorys, an impact bond was not necessary to fund these new processes: ‘similar initiatives have and are being delivered by ICRC without a HIB’ (Lau, 2021, p. 19). Rather than any specific features of the impact bond mechanism, predictable long‐term funding secured through the HIB enabled the programme results. This, though, could have been achieved via a multi‐year grant or the ICRC's annual appeals with careful management of financial risks (Interviewees 8 and 26; see also Lau, 2021).

The stated rationale for financing the RIB through an impact bond was to transfer to investors the risks inherent in livelihood programming, particularly given the limited evidence that programme inputs reliably generate tangible livelihood outcomes (Marköö, 2024). However, these risks were minimised in practice through a careful selection of the implementing partner: KOIS chose NEF from among 30 organisations delivering livelihood programmes in the region based on the strength of its intervention model, positive track record, and performance management capabilities (Convergence, 2017; Bery, 2023). Additionally, each outcome funder and investor undertook their own due diligence of NEF and vetted every aspect of the organisation, including programmes, operations, financial management, and governing board (Interviewee 15). As a result, the RIB had a good probability of success from the start, given NEF's expertise and organisational capacity.

The RIB's bespoke approach is regarded as its key innovation, enabling tailored training and mentorship aligned with individual needs and local market dynamics, unlike traditional input‐based models that typically rely on standardised interventions (Interviewee 5). The RIB evaluation also identifies adaptive management as a major strength: participants in later cohorts reported improvements in training content, flexibility, accessibility, and communication, reflecting iterative learning over time (Meyer, Borkum, and Collins, 2025). While these adaptations are not groundbreaking, they nonetheless represent a programme innovation in how services are delivered to recipients. At the same time, the evaluation underscores the need for further tailoring to address the context‐specific barriers faced by the most vulnerable subgroups, including refugees and women (Meyer, Borkum, and Collins, 2025).

Furthermore, the RIB's adaptive management and bespoke approach is enabled by predictable multi‐year funding and flexible features of outcome‐based financing, such as not having to implement prescribed activities and spend strictly as budgeted—this can occur without the involvement of investors. As one interviewee noted, the RIB's main outcome funders—the IKEA and Novo Nordisk Foundations—issue multi‐year funding and provide significant flexibility to their implementing partners as they are not bound by rigid structures and compliance hurdles in the same way as bilateral and multilateral government donors are (Interviewee 16). Therefore, financing the same programme through simpler alternatives, such as a flexible multi‐year grant or a hybrid model whereby a portion of the budget is tied to the achievement of outcomes, would likely have delivered similar results. This type of hybrid model would also address the liquidity concern that without investors providing the initial capital, smaller implementing organisations would not be able to pre‐finance their activities due to limited cash flow (Interviewee 21). In other words, there are simpler and cost‐effective funding models that can unlock similar benefits.

Overall, neither the HIB nor the RIB transferred substantial or novel risk to investors. The HIB involved routine ICRC activities with limited programme innovation, and the risks were primarily external rather than performance‐based. The RIB introduced modest programme innovations, but the selection of a capable implementing partner and thorough due diligence minimised performance risk from the outset. These findings support the SIB literature, which argues that impact bonds often diverge from the ideal model by avoiding the core features that were meant to make them advantageous, such as risk transfer and innovation (see, for example, Arena et al., 2016). Specifically, impact bonds do not fully transfer risk as investors often use derisking strategies, such as selecting service providers with a proven track record of delivering similar results, sharing potential loss with service providers, releasing the investment in tranches based on satisfactory performance, and ensuring their principal is fully or partially protected regardless of outcomes (Liang, Mansberger, and Spieler, 2014; Burand, 2019; Economy, Carter, and Airoldi, 2023). Furthermore, the Brookings Institution's analysis of 38 SIBs active in 2015 shows that there was ‘not a single intervention that [had] never been used before this SIB transaction’ (Gustafsson‐Wright, Gardiner, and Putcha, 2015, p. 43), illustrating that impact bonds generally fund tried and tested approaches rather than truly innovative approaches to existing problems—thereby negating the ‘innovation’ argument.

## 7 CONCLUSION

The first applications of impact bonds in humanitarian and refugee response were presented as a groundbreaking advance—a ‘paradigm shift’ (IKEA Foundation et al., 2021) and a ‘radical transformation’ (ICRC, 2017) in how aid is financed and delivered. By linking payment to the achievement of outcomes and transferring financial risk to investors, impact bonds were expected to mobilise new resources, improve aid effectiveness, and foster programme innovation. This article demonstrates that two pilot projects, the HIB and the RIB, produced more modest and mixed results than early promises suggested.

Although the rhetoric surrounding the adoption of impact bonds was closely tied to addressing the humanitarian funding gap, the cases examined here did not expand the overall pool of resources. Investors provided temporary financing rather than new funding and were repaid from the existing humanitarian budgets of government donors and foundations. These impact bonds thus served as a *financing* mechanism rather than a *funding* solution: they altered the flow of funds without increasing the volume of resources for crisis‐affected populations. In addition, they did not function as frontloading instruments to generate liquidity that was otherwise unavailable; instead of pledging future commitments, outcome funders pre‐positioned the required payments to assure investors.

While the impact bonds examined did not increase the overall quantity of humanitarian funding, they offered quality funding relative to annual grants or appeals. The four‐to‐five‐year predictable funding enabled the ICRC to invest in longer‐term physical and digital infrastructure development and allowed NEF to run its livelihoods programme adaptively across multiple cohorts. However, the HIB's programme design—centred on linear infrastructure interventions highly exposed to external risks—proved poorly suited to an outcome‐based financing instrument meant to incentivise iteration, adaptation, and management of performance risk. As most of the implementation period was absorbed by construction and set up, the physical rehabilitation centres achieved limited efficiency gains, far below target. Additionally, the choice of the programme and outcome metric was driven more by the structural and financial requirements of the impact bond mechanism than by opportunities to improve outcomes for affected populations. By contrast, the RIB made fuller use of the instrument's flexibility, applying adaptive management and learning across cohorts. This contributed to strong short‐ and medium‐term business survival but did not result in substantially better livelihood outcomes than comparable traditionally financed programmes. Despite its explicit focus on refugees, the RIB produced no statistically significant improvement in refugee household consumption, with benefits accruing primarily to host community members and relatively better‐off households.

Moreover, the impact bonds did not function as a genuine risk‐sharing mechanism, since investors' capital was protected by generous guarantees, and programmes did not involve significant innovation or unique performance risk necessitating investor involvement. When outcome funders are the same humanitarian donors that directly fund aid agencies—and who already accept significant risks owing to the volatile nature of humanitarian programmes—impact bonds hold limited value unless they empower implementers to try different approaches that they cannot do with conventional funding. As noted above, many of their positive effects stemmed from predictable multi‐year funding and adaptive management—characteristics not unique to impact bonds. Taken together, the evidence from the two cases suggests that the impact bond model offered limited advantages over simpler financing arrangements such as multi‐year grants, conventional PbR schemes, or hybrid performance‐based grants.

These findings align with critiques from the wider impact bond literature, which similarly highlight limited evidence that impact bonds deliver better outcomes than alternative financing arrangements and note that the degree of risk transferred to investors is often minimal. In the cases examined here, the absence of distinctive added value raises questions about whether the additional transaction costs, the administrative complexity, and the years spent structuring these instruments were justified. The article therefore underscores the importance of carefully assessing new financing tools against their stated aims, their design choices, and the specific constraints of crisis‐affected environments.

## CONFLICT OF INTEREST STATEMENT

The author declares no conflict of interest.

## Data Availability

Research data are not shared.

## Endnotes

^1^
Interviewees are referred to by numbered pseudonyms (1, 2, 3, etcetera) in a consistent but non‐identifying manner. See section three for more information.

^2^
Outputs are the immediate results of programme activities, often tangible deliverables or services produced by the programme (such as the amount of food distributed). Outcomes are longer‐term changes or improvements experienced by aid recipients as a result of the programme's outputs (such as improved food security). Inputs are the resources and contributions needed to achieve programme outputs, including human resources, equipment, and materials (such as amount of food procured).
